# Insulin resistance, clinical presentation and resistance to selective serotonin and noradrenaline reuptake inhibitors in major depressive disorder

**DOI:** 10.1007/s43440-024-00621-5

**Published:** 2024-07-09

**Authors:** Anna J. Krupa, Adrian A. Chrobak, Zbigniew Sołtys, Dominika Dudek, Bernadeta Szewczyk, Marcin Siwek

**Affiliations:** 1 Department of Affective Disorders, Jagiellonian University Medical College, ul. Kopernika 21a, Krakow, 31-501 Poland; 2 Department of Adult Psychiatry, Jagiellonian University Medical College, Kopernika 21a, Krakow, 31-501 Poland; 3 Institute of Zoology and Biomedical Research, Laboratory of Experimental Neuropathology, Jagiellonian University, Gronostajowa 9, Krakow, 30-387 Poland; 4 Department of Neurobiology, Maj Institute of Pharmacology, Polish Academy of Sciences, Smętna 12, Krakow, 31-343 Poland

**Keywords:** Major depressive disorder (MDD), Insulin resistance, Insulin, Depression subtype, Metabolic depression, Selective serotonin and noradrenaline reuptake inhibitors (SNRI)

## Abstract

**Background:**

The understanding of mechanisms underlying non-response to antidepressants is limited. The latest data highlights the role of insulin resistance (IR) in major depressive disorder (MDD) pathophysiology, presentation, and treatment efficacy. This work aimed to assess IR in MDD and explore the relationships between IR, MDD presentation and non-response to selective serotonin and noradrenaline reuptake inhibitors (SNRI).

**Methods:**

67 MDD individuals: 36 responsive (MDD T[+]), 31 non-responsive (MDD T[−]) to SNRI and 30 healthy controls were recruited. The treatment response criteria were: Clinical Global Impression Scale-Improvement score of 1 or 2 after ≥ 8 weeks of treatment. Participants were assessed by physician and self-report tools measuring depression, anhedonia, anxiety, bipolarity, sleep quality. Blood samples were collected to assess fasting glucose and insulin levels and calculate HOMA-IR (homeostasis model assessment of insulin resistance).

**Results:**

MDD T[-] vs. MDD T[+] had significantly higher body mass index, insulin levels, and HOMA-IR. MDD T[-] presented higher levels of depressed mood, appetite/weight changes, loss of interest, energy, overall depressive symptoms, and sleep impairment; some evaluations suggested higher anhedonia and anxiety in MDD T[-] vs. MDD T[+]. Insulin and IR were weakly but significantly correlated with the severity of psychomotor symptoms, energy level, thoughts of death/suicide, self-criticism, appetite/weight, depressed mood symptoms, sleep problems. IR was weakly but significantly correlated with anhedonia.

**Conclusion:**

IR appears to be linked to depressive symptoms characteristic of the “metabolic” MDD subtype, such as psychomotor changes, energy level, anhedonia, sleep problems, appetite/weight changes, state and trait anxiety, sleep quality, and non-response to SNRI.

## Introduction

Globally, major depressive disorder (MDD) has been among the top causes of disability for the past 30 years and its impact has risen even higher due to the COVID-19 pandemic. Despite this pervasive influence that MDD has on the worldwide burden of disease, its treatment efficacy remains limited as only 50–60% of patients respond to first-line pharmacotherapy, and the percentage of responders drops with each consequent treatment attempt [1–4]. Hence, it is vital to expand our comprehension of MDD and its management. These days, there is a wide range of available antidepressant treatment options which were proven effective. Yet there is a paucity of data on the factors associated with the response to specific antidepressants or lack thereof. Most of the available MDD treatment guidelines suggest selective serotonin reuptake inhibitors (SSRI) as the drugs of first choice. Others also pose selective serotonin and noradrenaline reuptake inhibitors (SNRI), bupropion, mirtazapine, vortioxetine, and agomelatine as first-choice options. The majority of guidelines suggest that the antidepressant choice should be based on the dimensional assessment of MDD clinical presentation, and there is some data supporting the use of specific antidepressant drugs in particular MDD subtypes as classified by the Diagnostic and Statistical Manual of Mental Disorders-5th edition (DSM-5) [5, 6]. However, little is known about which pathophysiological mechanisms are related to response or non-response to specific antidepressants.

Recently, evidence has accumulated to support the significant role of insulin resistance (IR) in MDD [7]. Firstly, it was shown that insulin has a direct impact on serotoninergic and dopaminergic neurotransmission, which disruptions are well-known to be linked to depressive symptomatology [8, 9]. As reported in the studies focusing on serotoninergic pathways, insulin administration had anxiolytic effects and augmented the antidepressant action of fluoxetine [8]. At the same time, research focusing on dopaminergic pathways unraveled that insulin differently modulates dopamine release in the ventral tegmental area and nucleus accumbens [10–12] and that insulin administered intranasally changes the activity and connectivity of dopaminergic circuits and alters reward behavior [9, 13]. In addition, animal and human studies imply that the insulin modulation of serotoninergic and dopaminergic systems is dysregulated in IR subjects [8, 14]. Furthermore, it was observed that IR was a state-marker of MDD, but that held true only for patients with atypical but not typical depression [15]. Moreover, associations between IR and specific depressive symptoms, that is: increased appetite/ weight, hypersomnia, anhedonia, fatigue, and irritability, were reported [16, 17]. What is more, evidence emerges that IR is linked to non-response to antidepressants. Rashidian et al. reported that in individuals with MDD treated with vortioxetine, changes in IR mediated the improvement of depression [18] and that IR was a negative predictor of the early change in anhedonia, reduction of cognitive and functional impairment as well as response to vortioxetine [19]. In our previous works, we have noted that IR was a predictor of lack of response to SNRI in fibromyalgia (58% of the studied group was comorbid with depression) [20] and that psychological variables [21], as well as psychopathological symptoms such as the presence of depression, anxiety, anhedonia [22], cognitive impairments and diurnal rhythm disruptions were related to non-response to SNRI in fibromyalgia [23, 24]. Also, in the pilot analysis of this work, we showed that in subjects with MDD, higher IR was linked to non-response to SNRI [25]. The aim of this study was to assess IR in MDD patients and explore the relationships between IR and MDD clinical presentation as well as non-response to SNRI.

## Materials and methods

### Recruitment and participants

This was a cross-sectional study performed between January and June 2023; this study was part of a larger project assessing the factors associated with response to SNRI in patients with various diagnoses (not solely MDD but also chronic pain patients). Participants were recruited from the inpatient and outpatient psychiatric wards and clinic of the Clinical Department of Psychiatry of the University Hospital in Krakow, Poland. The inclusion criteria for the patient groups were as follows: (1) diagnosis of MDD according to the International Statistical Classification of Diseases-10th revision and DSM-5, (2) age 18–65, (3) history of treatment with SNRI duloxetine (60–120 mg/d) or venlafaxine (150–225 mg/d) of ≥ 8 weeks duration. The exclusion criteria for the patient groups were: (1) diagnosis of diabetes mellitus, (2) any other severe, acute, or chronic neurological or other somatic disorders, (3) substance use disorder (SUD)(other than nicotine), (4) history of psychotic symptoms, (5) diagnosis of schizophrenia spectrum disorder or bipolar disorder, (6) no history of SNRI treatment or history of taking subtherapeutic SNRI doses or history of taking an SNRI for < 8 weeks. MDD patients were classified as responsive to SNRI (MDD T[+]) or non-responsive to SNRI (MDD T[-]). The data on treatment response were based on Clinical Global Impression Scale (CGI)(a standard evaluation performed by our team of attending physicians during follow-up visits and documented in the clinical records). The criteria of treatment response were: Clinical Global Impression Scale-Improvement (CGI-I) score of 1 or 2 (“Very Much Improved” or “Much Improved”) as evaluated by the attending physician after ≥ 8 weeks of treatment. The choice of a specific SNRI (duloxetine vs. venlafaxine), the dose and its’ adjustments was up to the attending physician who preceded these decisions with careful consideration of potential contraindications, drug interactions, treatment effects and tolerance.

Furthermore, a group of healthy controls (HC) was recruited. The inclusion criteria for the HC were: 1) age 18–65. The exclusion criteria for the HC were: (1) any severe, acute, or chronic psychiatric disorders, (2) any severe, acute, or chronic somatic disorders, and (3) SUD (other than nicotine). All HCs were interviewed and examined by a physician to rule out any diseases.

In both the MDD and HC groups, we included individuals with well-controlled asthma, allergies, dermatoses, thyroid insufficiency, hyperlipidemia, and hypertension. All subjects were thoroughly interviewed and examined by a psychiatrist, and if needed, further consultations and tests were performed to rule out any serious comorbidities.

### Laboratory assessments

Venous blood samples were collected from participants after at least 12 h of fasting. The assessments included serum levels of glucose and insulin. The tests were executed by a certified diagnostic laboratory. Based on the obtained data, the HOMA-IR (homeostasis model assessment of insulin resistance) was calculated to measure the level of IR [26].

### Psychopathological assessments

Each subject filled Polish adaptations of self-report tools to evaluate:

1. depression- the self-rated Quick Inventory of Depressive Symptomatology (QIDS), Hospital Anxiety and Depression Scale (HADS)- depression subscale (HADS-D),
2. hedonic tone- the Snaith–Hamilton Pleasure Scale (SHAPS) and the Dimensional Anhedonia Rating Scale (DARS),
3. bipolar spectrum features- the Mood Disorder Questionnaire (MDQ), Hypomania Checklist (HCL),
4. state and trait anxiety- the State and Trait Anxiety Inventory (STAI), HADS- anxiety subscale (HADS-A),
5. sleep quality- the Pittsburgh Sleep Quality Index (PSQI).

### Study sample

Initially, 105 MDD subjects were recruited for the study. However, 27 were not enrolled because the observation and diagnostic process revealed serious comorbidities (such as bipolar disorder, emergence of psychotic symptoms, SUD, diabetes mellitus, or others), and 11 did not consent to participate. Among those not enrolled due to serious somatic comorbidities, 15 were non-responsive to SNRI, and 12 were responsive to SNRI. Among those not enrolled due to the lack of consent to participate in the study, 7 were non-responsive to SNRI, and 4 were responsive to SNRI.

All participants provided informed written consent. The study was approved by the local Bioethical Committee (No. 1072.6120.276.2022).

### Statistical analysis

Statistical analyses were performed using R software [27] and functions from rstatix, psych, and stats package. For quantitative data, t-test or one-way analysis of variance (ANOVA), with corrections for nonhomogeneous variances in the cases of significant Levene’s test. For pairwise comparisons after ANOVA the Games-Howell test was used. For qualitative data, the Chi-squared test was used. *P*-values lower than 0.05 were considered significant. Moreover, *p*-values obtained from t-test or ANOVA were corrected using the Benjamini-Yekutieli procedure. For the visualization of the results, ggplot2 and ggcorrplot packages were used [28].

## Results

### Group characteristics

97 subjects participated in this study, 36 MDD T[+], 31 MDD T[-] and 30 HC. All groups were comparable with regard to age and the proportion of sexes, subjects with hypertension, thyroid insufficiency, and those who smoked. MDD T[-] had a higher body mass index (BMI) compared to HC and MDD T[+], while no differences were observed between HC and MDD T[+] or HC and MDD as a whole group. The proportion of subjects with hyperlipidemia was higher in MDD T[-] vs. MDD T[+], but no differences in proportions of individuals with hyperlipidemia were noted in MDD T[+] or MDD T[-] vs. HC or MDD T[+] vs. HC (Table 1).

**Table 1:** Basic characteristics of the researched group* (healthy controls, patients with major depressive disorder responsive or non-responsive to treatment with serotonin and noradrenaline reuptake inhibitors): age, sex, BMI, comorbidities, smoking status

Variable	HC *n* = 30	MDD *n* = 67	MDD T [+] *n* = 36	MDD T [-] *n* = 31	HC vs. MDD T [+] vs. MDD T [-] ^1^	HC vs. MDD T[+]	HC vs. MDD T[-]	MDD T [+] vs. MDD T [-]
Age mean years ± SD	44.50 ± 12.37	42.97 ± 13.52	42.72 ± 12.42	43.26 ± 14.90	F_2, 94_ = 0.152 *p* = 0.86 p.adj > 0.99	*p* = 0.85	*p* = 0.93	*p* = 0.98
Sex (female)	27	53	32	21	χ2 = 6.855 df = 2 *p* = 0.03 p.adj = 0.18	*p* > 0.99	*p* = 0.07	*p* = 0.07
BMI kg/m2 ± mean	23.96 ± 3.74	25.57 ± 4.91	23.86 ± 3.78	27.54 ± 5.36	F_2, 94_ = 7.424 *p* = 0.001 **p.adj = 0.007**	*p* > 0.99	*p* = 0.005	*p* = 0.002
Hyperlipidemia (yes)	4	12	1	11	χ2 = 13.251 df = 2 *p* = 0.001 **p.adj = 0.007**	*p* = 0.25	*p* = 0.09	*p* = 0.002
Hypertension (yes)	5	9	2	7	χ2 = 4.085 df = 2 *p* = 0.13 p.adj = 0.72	*p* = 0.29	*p* = 0.80	*p* = 0.09
Hypothyroidism (yes)	2	8	4	4	χ2 = 0.681 df = 2 *p* = 0.71 p.adj > 0.99	*p* = 0.84	*p* = 0.70	*p* > 0.99
Smoking (yes)	2	15	9	6	χ2 = 3.911 df = 2 *p* = 0.14 p.adj = 0.75	*p* = 0.10	*p* = 0.28	*p* = 0.80

BMI- body mass index, df = degrees of freedom, HC- healthy controls, MDD- major depressive disorder, MDD T [+]- patients responsive to SNRI treatment, MDD T [-]- patients non-responsive to SNRI treatment, p.adj - p adjusted for multiple comparisons (Benjamini-Yekutieli method), SD- standard deviation, SNRI- selective serotonin and noradrenalin reuptake inhibitors

χ2 test was used to compare the qualitative data. ^1^analysis of variance (ANOVA) was used to assess the differences in quantitative data with following Tukey test for multiple comparisons

*major depressive disorder patients treated in the Clinical Department of Psychiatry of the University Hospital in Krakow, Poland between January and June 2023 and a healthy control group

### Metabolic variables

**Fig. 1:**
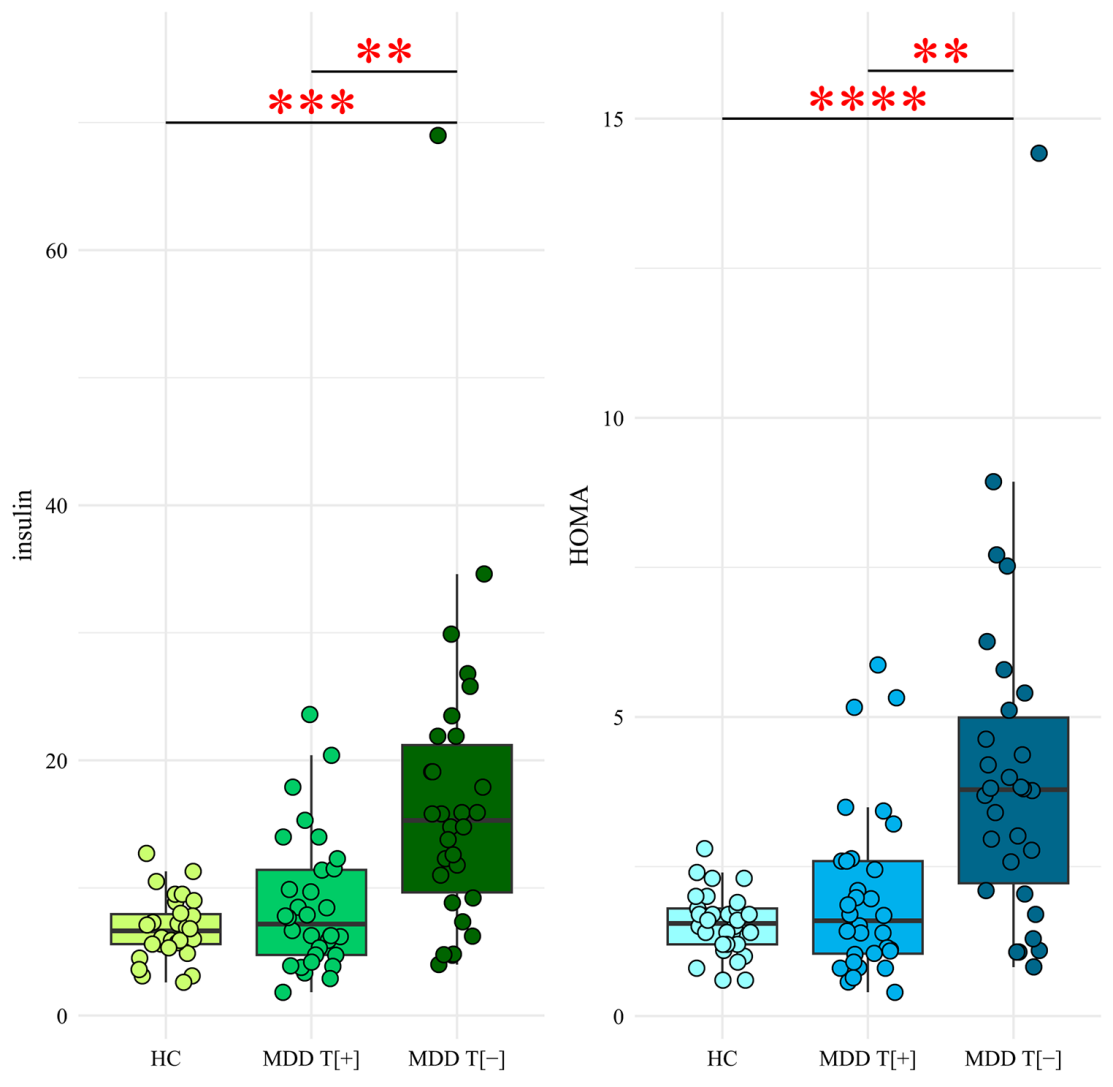
Comparisons of fasting insulin levels and HOMA-IR in the researched group* (healthy controls, patients with major depressive disorder responsive or non-responsive to treatment with serotonin and noradrenaline reuptake inhibitors) *major depressive disorder patients treated in the Clinical Department of Psychiatry of the University Hospital in Krakow, Poland between January and June 2023 and a healthy control group **p < 0.01, ***p < 0.001; ****p < 0.0001, Games-Howell test for multiple comparisons Insulin was reported in [uU/mL]. Boxplots show spread of the data. The line within the box represents the median, the lower and upper parts of the box represent the second and third quartiles, and the whiskers reach 1.5 times the interquartile range. Dots beyond the whiskers represent outlier values HC- healthy controls (*n* = 30), MDD T [+]- patients responsive to SNRI treatment (*n* = 36), MDD T[-] - patients non-responsive (*n* = 31)

No significant differences in the mean fasting glucose levels were observed among the studied groups. MDD T[-] showed higher mean fasting insulin levels than HC and MDD T[+]. MDD T[-] presented higher HOMA-IR than HC and MDD T[+] (Table 2; Fig. 1).

**Table 2:** Comparisons of fasting glucose and fasting insulin levels and HOMA-IR in the researched group* (healthy controls, patients with major depressive disorder responsive or non-responsive to treatment with serotonin and noradrenaline reuptake inhibitors)

Group	HC *n* = 30	MDD *n* = 67^1^	MDD T[+] *n* = 36^2^	MDD T[-] *n* = 31^3^	HC vs. MDD T [+] vs. MDD T [-] ^3^	HC vs. MDD T[+]	HC vs. MDD T[-]	MDD T [+] vs. MDD T [-]
Metabolic variables								
Fasting glucose in mmol/l Mean (SD)	5.11 ± 0.42	5.30 ± 0.57	5.23 ± 0.62	5.38 ± 0.52	F_2, 89_ = 1.932 *p* = 0.15 p.adj = 0.76 η2 = 0.04 small	*p* = 0.64	*p* = 0.13	*p* = 0.52
Insulin in uU/mol Mean (SD)	6.81 ± 2.42	12.75 ± 10.33	8.64 ± 5.26	17.13 ± 12.51	F_2, 89_ = 10.487 **p, p.adj < 0.001** **η2 = 0.25 large**	*p* = 0.19	*p* < 0.001	*p* = 0.004
HOMA-IR Mean (SD)	1.55 ± 0.52	3.02 ± 2.44	2.00 ± 1.40	4.10 ± 2.83	F_2, 89_ = 12.34 **p, p.adj < 0.001 η2 = 0.27 large**	*p* = 0.22	*p* < 0.001	*p* = 0.002

^1^- analyses of insulin levels and HOMA-IR values were performed in 62 subjects, ^2^- analyses of insulin levels and HOMA-IR values were performed in 32 subjects, ^3^- analyses of insulin levels and HOMA-IR values were performed in 30 subjects, ^3^- ANOVA was used to assess the differences in quantitative data with the correction to nonhomogeneous variance, when necessary, and Games-Howell test for multiple comparisons, η2 - (eta squared) is the measure of effect size lower than 0.01 was counted as negligible, 0.01–0.06 as small, 0.06–0.14 as medium and higher than 0.14 as large

*major depressive disorder patients treated in the Clinical Department of Psychiatry of the University Hospital in Krakow (Poland) between January and June 2023 and a healthy control group

ANOVA- analysis of variance, HC- healthy controls, HOMA-IR- homeostatic model assessment of insulin resistance, MDD- major depressive disorder, MDD T [+]- patients responsive to SNRI treatment, MDD T [-]- patients non-responsive to SNRI treatment, SNRI- selective serotonin and noradrenaline reuptake inhibitors, p.adj - p adjusted for multiple comparisons (Benjamini-Yekutieli method), SD- standard deviation

### Psychopathology

#### Depression

**Fig. 2:**
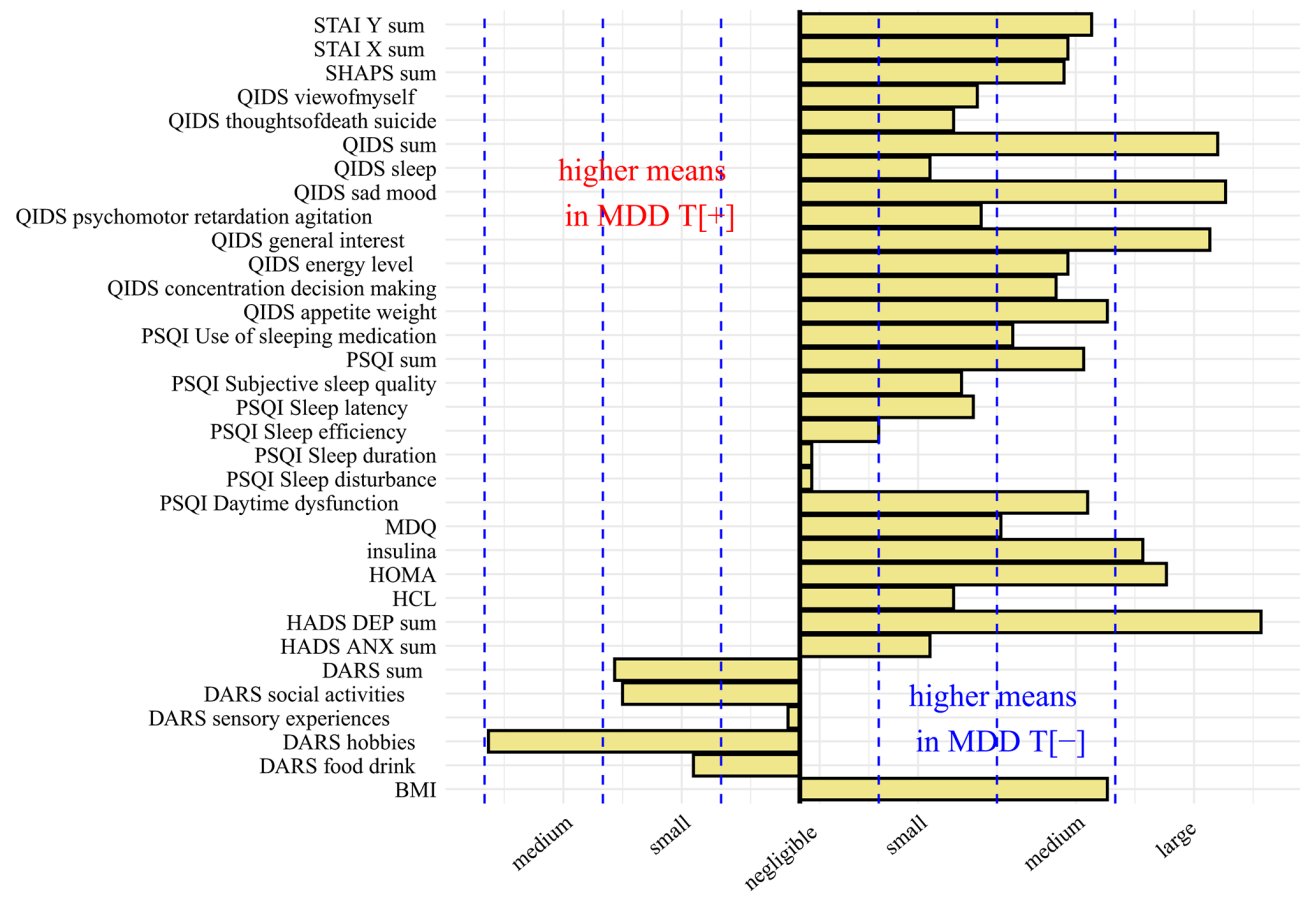
Effect sizes for comparisons of depression, anhedonia, anxiety, bipolarity, sleep quality, fasting insulin, HOMA-IR and BMI in patients with major depressive disorder responsive or non-responsive to treatment with serotonin and noradrenaline reuptake inhibitors* *major depressive disorder patients (*n* = 67) treated in the Clinical Department of Psychiatry of the University Hospital in Krakow, Poland between January and June 2023 The yellow bars visualize the Hedges’ g effect size BMI- body mass index, DARS- Dimensional Anhedonia Rating Scale, HADS-A- Hospital Anxiety and Depression Scale anxiety subscale, HADS-D- Hospital Anxiety and Depression Scale depression subscale, HCL- Hypomania Checklist, HOMA-IR- homeostasis model assessment of insulin resistance, MDD T [+]- patients responsive to SNRI treatment, MDD T [-]- patients non-responsive to SNRI treatment, MDQ- Mood Disorder Questionnaire, PSQI- Pittsburgh Sleep Quality Index, SHAPS- Snaith-Hamilton Pleasure Scale, STAI-X- State and Trait Anxiety Inventory state subscale, STAI-Y- State and Trait Anxiety Inventory trait subscale, QIDS- Quick Inventory of Depressive Symptomatology

The levels of depression were higher in MDD T[-] vs. HC as indicated by all QIDS items (apart from appetite/weight) total QIDS score as well as HADS-D. The comparison of MDD T[+] vs. HC showed higher QIDS sleep-related, concentration/ decision making, view of myself, energy level, psychomotor items, and total QIDS, as well as HADS-D scores. MDD T[-] vs. MDD T[+] presented higher QIDS feeling sad, appetite/ weight, general interest, energy level, total QIDS, and HADS-D scores (Table 3; Fig. 2).

**Table 3:** Comparisons depression, anhedonia, anxiety, bipolarity and sleep quality levels in the researched group* (healthy controls, patients with major depressive disorder responsive or non-responsive to treatment with serotonin and noradrenaline reuptake inhibitors)

Variable	HC mean score ± SD	MDD mean score ± SD	MDD T [+] mean score ± SD	MDD T [-] mean score ± SD	ANOVA effect size^1^	HC vs. MDD T [+]	HC vs. MDD T [-]	MDD T [+] vs. MDD T [-]
QIDS sleep	1.20 ± 0.96	1.96 ± 0.90	1.82 ± 0.98	2.12 ± 0.78	F_2, 80_ = 7.289 *p* = 0.001 **p.adj = 0.007** **η2 = 0.15** **large**	*p* = 0.03	*p* = 0.001	*p* = 0.47
QIDS feeling sad	0.37 ± 0.49	1.19 ± 0.96	0.75 ± 0.89	1.68 ± 0.80	F_2, 80_ = 22.326 **p, p.adj < 0.001** **η2 = 0.36** **large**	*p* = 0.12	*p* < 0.001	*p* < 0.001
QIDS appetite/ weight	0.80 ± 0.96	1.36 ± 1.13	0.96 ± 1.04	1.80 ± 1.08	F_2, 80_ = 7.258 *p* = 0.001 **p.adj = 0.007** **η2 = 0.15** **large**	*p* = 0.81	*p* = 0.001	*p* = 0.01
QIDS concentration/ decision making	0.30 ± 0.47	1.11 ± 0.97	0.82 ± 0.86	1.44 ± 1.00	F_2, 80_ = 14.936 **p, p.adj < 0.001** **η2 = 0.26** **large**	*p* = 0.02	*p* = 0.001	*p* = 0.053
QIDS view of myself	0.13 ± 0.57	1.09 ± 1.27	0.82 ± 1.25	1.40 ± 1.26	F_2, 80_ = 12.269 **p, p.adj < 0.001** **η2 = 0.20** **large**	*p* = 0.03	*p* < 0.001	*p* = 0.22
QIDS thoughts of death/ suicide^2^	-	-	0.32 ± 0.55	0.64 ± 0.99	t_51_= -1.465 *p* = 0.15 p.adj = 0.76 g = 0.39 small #	-	-	-
QIDS general interest	0.17 ± 0.46	0.70 ± 0.99	0.25 ± 0.44	1.20 ± 1.19	F_2, 80_ = 8.08 *p* = 0.001 **p.adj = 0.005** **η2 = 0.28** **large**	*p* = 0.76	*p* < 0.001	*p* = 0.002
QIDS energy level	0.20 ± 0.48	1.06 ± 0.99	0.75 ± 0.89	1.40 ± 1.00	F_2, 80_ = 16.226 **p, p.adj < 0.001** **η2 = 0.27** **large**	*p* = 0.02	*p* < 0.001	*p* = 0.04
QIDS psychomotor	0.20 ± 0.61	1.36 ± 1.00	1.14 ± 1.04	1.60 ± 0.91	F_2, 80_ = 23.811 **p, p.adj < 0.001** **η2 = 0.32** **large**	*p* < 0.001	*p* < 0.001	*p* = 0.21
QIDS sum	3.37 ± 2.11	10.30 ± 5.88	7.64 ± 4.81	13.28 ± 5.60	F_2, 80_ = 38.906 **p, p.adj < 0.001** **η2 = 0.47** **large**	*p* < 0.001	*p* < 0.001	*p* < 0.001
HADS-D sum	3.73 ± 2.35	7.76 ± 4.34	5.69 ± 3.40	10.16 ± 4.11	F_2, 81_ = 23.412 **p, p.adj < 0.001** **η2 = 0.39** **large**	*p* = 0.04	*p* < 0.001	*p* < 0.001
SHAPS continous sum	0.50 ± 1.17	2.44 ± 3.05	1.52 ± 2.31	3.52 ± 3.48	F_2, 81_ = 9.515 *p* < 0.001 **p.adj = 0.002** **η2 = 0.21** **large**	*p* = 0.01	*p* < 0.001	*p* = 0.048
DARS hobbies	12.93 ± 3.69	10.15 ± 4.03	11.57 ± 3.73	8.56 ± 3.82	F_2, 80_ = 9.584 *p* < 0.001 *p* = 0.002 **η2 = 0.19** **large**	*p* = 0.35	*p* < 0.001	*p* = 0.01
DARS foods/drinks	11.63 ± 4.02	10.06 ± 4.24	10.61 ± 4.40	9.44 ± 4.05	F_2, 80_ = 1.894 *p* = 0.16 p.adj = 0.78 η2 = 0.05 small	*p* = 0.62	*p* = 0.13	*p* = 0.57
DARS social activities	12.23 ± 2.28	8.43 ± 4.35	9.36 ± 4.24	7.40 ± 4.33	F_2, 80_ = 14.548 **p, p.adj < 0.001** **η2 = 0.23** **large**	*p* = 0.008	*p* < 0.001	*p* = 0.23
DARS sensory experiences	12.37 ± 2.51	9.79 ± 4.27	9.86 ± 4.30	9.72 ± 4.32	F_2, 80_ = 4.505 *p* = 0.01 p.adj = 0.06 η2 = 0.23 large	*p* = 0.03	*p* = 0.03	*p* = 0.99
DARS sum	49.17 ± 9.12	38.43 ± 13.57	41.39 ± 14.33	35.12 ± 12.09	F_2, 80_ = 9.513 ***p*** **< 0.001 p.adj = 0.002** **η2 = 0.19** **large**	*p* = 0.04	*p* < 0.001	*p* = 0.14
STAI-X sum	34.50 ± 7.99	44.35 ± 14.09	40.10 ± 15.04	49.28 ± 11.29	F_2, 80_ = 10.821 **p, p.adj < 0.001** **η2 = 0.21** **large**	*p* = 0.17	*p* < 0.001	*p* = 0.01
STAI-Y sum	41.70 ± 7.66	50.76 ± 12.49	46.72 ± 14.04	55.44 ± 8.49	F_2, 81_ = 11.78 **p, p.adj < 0.001** **η2 = 0.26** **large**	*p* = 0.16	*p* < 0.001	*p* = 0.009
HADS-A sum	6.27 ± 3.40	8.50 ± 4.12	7.86 ± 4.22	9.24 ± 3.94	F_2, 81_ = 4.08 *p* = 0.02 p.adj = 0.12 η2 = 0.09 medium	*p* = 0.26	*p* = 0.01	*p* = 0.39
MDQ sum	2.03 ± 2.08	4.05 ± 3.37	3.25 ± 3.11	4.96 ± 3.47	F_2, 87_ = 7.264 *p* = 0.001 **p.adj = 0.007** **η2 = 0.014** **medium**	*p* = 0.24	*p* < 0.001	*p* = 0.07
HCL sum	10.4 ± 6.02	11.3 ± 7.10	10.00 ± 6.84	12.79 ± 7.23	F_2, 87_ = 1.469 *p* = 0.24 p.adj > 0.99 η2 = 0.03 small	*p* = 0.97	*p* = 0.37	*p* = 0.25
PSQI subjective sleep quality	0.77 ± 0.57	1.13 ± 0.68	1.00 ± 0.61	1.28 ± 0.74	F_2, 80_ = 4.435 *p* = 0.01 p.adj = 0.06 η2 = 0.1 small	*p* = 0.35	*p* = 0.01	*p* = 0.25
PSQI sleep latency	0.73 ± 0.64	1.39 ± 0.90	1.21 ± 0.86	1.60 ± 0.91	F_2, 81_ = 7.975 *p* < 0.001 **p.adj = 0.006** **η2 = 0.16** **large**	*p* = 0.07	*p* < 0.001	*p* = 0.18
PSQI sleep duration	0.60 ± 0.81	1.02 ± 1.45	1.00 ± 0.98	1.04 ± 1.86	F_2, 80_ = 1.056 *p* = 0.35 p.adj > 0.99 η2 = 0.03 small	*p* = 0.45	*p* = 0.41	*p* = 0.99
PSQI sleep efficicency	0.33 ± 0.61	0.58 ± 0.91	0.50 ± 1.00	0.68 ± 0.80	F_2, 80_ = 1.235 *p* = 0.3 p.adj > 0.99 η2 = 0.03 small	*p* = 0.72	*p* = 0.26	*p* = 0.70
PQSI sleep disturbance	0.90 ± 0.31	1.11 ± 0.42	1.11 ± 0.42	1.12 ± 0.44	F_2, 80_ = 2.905 *p* = 0.06 p.adj = 0.34 η2 = 0.07 medium	*p* = 0.11	*p* = 0.1	*p* = 0.99
PSQI sleep medication use	0.13 ± 0.57	1.13 ± 1.36	0.79 ± 1.23	1.52 ± 1.42	F_2, 80_ = 11.931 **p, p.adj < 0.001** **η2 = 0.21** **large**	*p* = 0.04	*p* < 0.001	*p* = 0.12
PSQI daytime disfunction	1.13 ± 0.86	2.63 ± 1.52	2.14 ± 1.43	3.20 ± 1.44	F_2, 81_ = 18.323 **p, p.adj < 0.001** **η2 = 0.31** **large**	*p* = 0.008	*p* < 0.001	*p* = 0.008
PSQI sum	4.60 ± 2.59	8.91 ± 4.11	7.59 ± 3.97	10.44 ± 3.80	F_2, 81_ = 19.259 **p, p.adj < 0.001** **η2 = 0.32** **large**	*p* = 0.004	*p* < 0.001	*p* = 0.009

^1^- ANOVA was used to assess the differences in quantitative data, η2 - (eta squared) is the measure of effect size effect size lower than 0.01 was counted as negligible, 0.01–0.06 as small, 0.06–0.14 as medium and higher than 0.14 as large. ^2^T-test was used to assess the differences in quantitative data, g- Hedges g is the measure of effect size. Effect size lower than 0.2 was counted as negligible. 0.2–0.5 as small. 0.5–0.8 as medium and for 0.8 as large, #- no comparison was made for the thoughts of death/ suicide item, because no HC scored higher than 0 on this item

ANOVA- analysis of variance, DARS- Dimensional Anhedonia Rating Scale, HADS-A- Hospital Anxiety and Depression Scale anxiety subscale, HADS-D- Hospital Anxiety and Depression Scale depression subscale, HC- healthy controls, HCL- Hypomania Checklist, HOMA-IR- homeostatic model assessment of insulin resistance, MDD- major depressive disorder, MDD T [+]- patients responsive to SNRI treatment, MDD T [-]- patients non-responsive to SNRI treatment, MDQ- Mood Disorder Questionnaire, p.adj - p adjusted for multiple comparisons (Benjamini-Yekutieli method), PSQI- Pittsburgh Sleep Quality Index, SD- standard deviation, SHAPS- Snaith-Hamilton Pleasure Scale, SNRI- selective serotonin and noradrenalin reuptake inhibitors, STAI-X- State and Trait Anxiety Inventory state subscale, STAI-Y- State and Trait Anxiety Inventory trait subscale, QIDS- Quick Inventory of Depressive Symptomatology,

#### Anhedonia

Both MDD T[+] and MDD T[-] presented higher anhedonia as evaluated by SHAPS total score, DARS subscales of social activities, and DARS total score vs. HC. MDD T[-] also showed lower hedonia as assessed with DARS hobbies subscale scores than HC. Compared to MDD T[+], MDD T[-] revealed higher anhedonia as measured by the total SHAPS score and DARS subscales of hobbies (Table 3; Fig. 2).

#### Anxiety

MDD T[-] presented higher levels of anxiety as evaluated by STAI state and trait subscales (but not HADS) vs. HC. No differences in the severity of anxiety were found between MDD T[+] and HC groups. MDD T[-] had higher anxiety than MDD T[+] as rated with STAI state and trait subscales but not HADS (Table 3; Fig. 2).

#### Bipolarity

The comparison between subgroups indicated that MDD T[-] had higher levels of bipolar spectrum features than HC as assessed by MDQ but not HCL. No other significant differences in levels of bipolar spectrum features were found between the studied groups (Table 3; Fig. 2).

#### Sleep quality

Both MDD T[+] and MDD T[-] presented higher sleep medication use, daytime dysfunction, and total PQSI scores than HC. MDD T[-] vs. MDD T[+] had higher daytime dysfunction and total PQSI score (Table 3; Fig. 2).

### Associations between insulin resistance (IR) and psychopathological variables

**Fig. 3:**
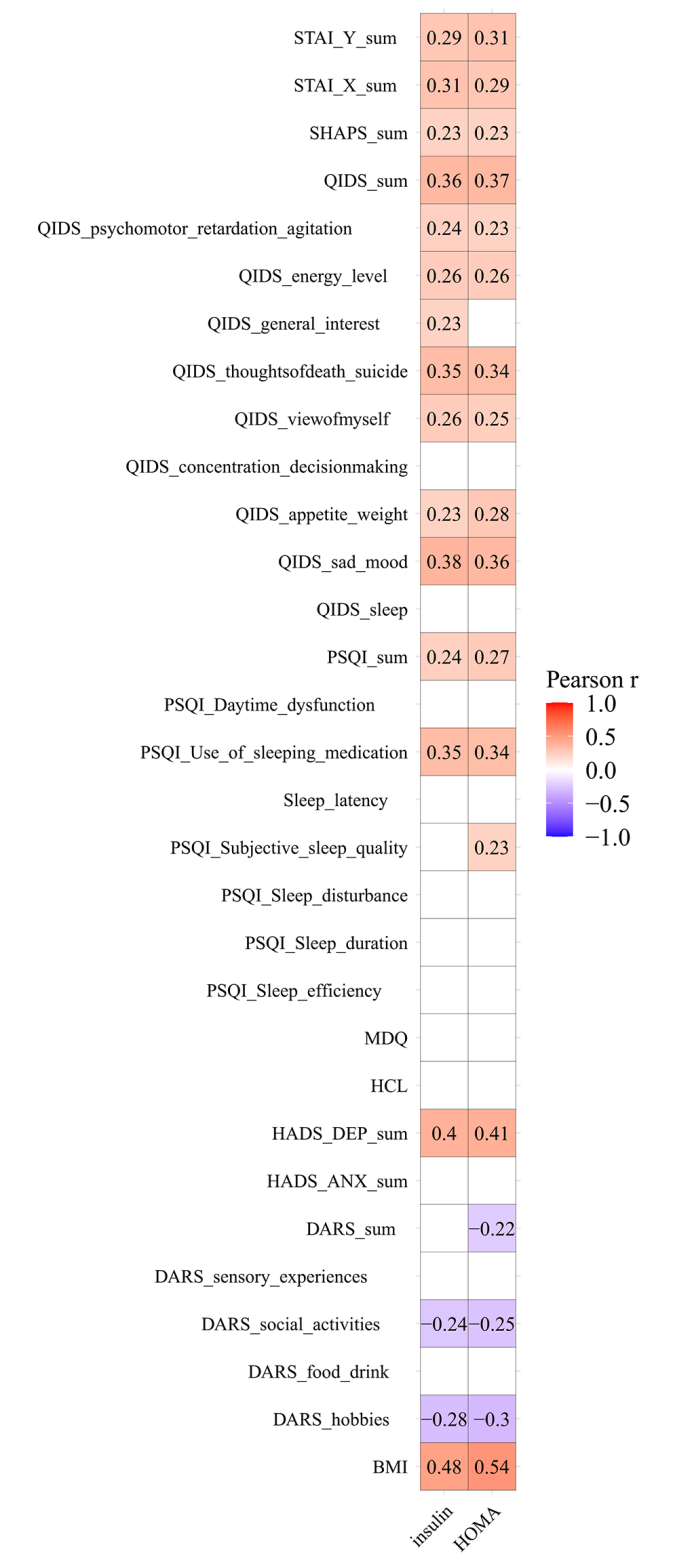
Correlations between insulin, HOMA-IR and levels of depression, anhedonia, anxiety, bipolarity, sleep quality in patients with major depressive disorder* *major depressive disorder patients (*n* = 67) treated in the Clinical Department of Psychiatry of the University Hospital in Krakow, Poland between January and June 2023 Only significant correlations are shown BMI- body mass index, DARS- Dimensional Anhedonia Rating Scale, HADS-A- Hospital Anxiety and Depression Scale anxiety subscale, HADS-D- Hospital Anxiety and Depression Scale depression subscale, HCL- Hypomania Checklist, HOMA-IR- homeostasis model assessment of insulin resistance, MDQ- Mood Disorder Questionnaire, PSQI- Pittsburgh Sleep Quality Index, SHAPS- Snaith-Hamilton Pleasure Scale, STAI-X- State and Trait Anxiety Inventory state subscale, STAI-Y- State and Trait Anxiety Inventory trait subscale, QIDS- Quick Inventory of Depressive Symptomatology

Correlation analysis performed in the MDD group as a whole has shown that the following parameters were significantly associated with the levels of insulin and IR: (1) depression as measured with QIDS psychomotor, energy level, general interest (the link was only significant for insulin but not IR), thoughts of death/ suicide, view of myself, appetite/ weight, feeling sad and total score as well as HADS-D sum, (2) anhedonia as rated with total SHAPS, DARS hobbies, social activities and DARS sum (the link to DARS sum was only significant for IR but not insulin), (3) anxiety as evaluated with STAI state and trait subscales (but not HADS-A), (4) sleep as measured with PSQI subjective sleep quality (the link was only significant for IR but not insulin), use of sleeping medication components and total score (Fig. 3). No significant correlations were found between BMI and any of the psychopathological dimensions.

## Discussion

The results showed that MDD T[-] had significantly higher BMI, fasting insulin, and IR than MDD T[+] and HC. The comparison of MDD T[+] and HC regarding fasting insulin and IR yielded no significant differences. Our observations are in line with those reported in the meta-analysis by Fernandes et al., which indicated that insulin and IR (but not fasting glucose) are higher in acute MDD subjects receiving antidepressants. Similarly, we found no differences in fasting glucose levels in MDD participants responsive or non-responsive to pharmacotherapy. However, contrary to their work, we have noted that MDD subjects non-responsive to SNRI MDD T[-] presented higher fasting insulin and IR than MDD individuals responsive to SNRI MDD T[+]. Their analyses were based on relatively small and heterogeneous groups of MDD individuals from 9 prospective studies who received different drugs with varying duration of treatment, in which baseline and post-pharmacotherapy metabolic data was available [15]. Also, while in the meta-analysis, these works were classified as offering data on responsive and non-responsive individuals, all but one either included numerical changes in pre and post-treatment scores of depression rating tools or reported the results for remitters vs. non-remitters instead of responders vs. non-responders. In particular Kahl et al. noted no differences between remitters vs. non-remitters in insulin or HOMA-IR levels after different antidepressant treatments (*n* = 37); Pinar et al. reported that insulin and HOMA-IR significantly increased while depression severity decreased after maprotiline treatment (*n* = 34); Moosa et al. showed that in responders insulin and HOMA-IR increased after fluoxetine treatment (*n* = 11) but decreased after imipramine treatment (*n* = 7); Chang et al. found no differences in insulin and HOMA-IR in responders and non-responders to venlafaxine or fluoxetine (*n* = 50); Kauffman et al. reported comparable insulin and HOMA-IR in citalopram responders vs. non-responders (*n* = 14); Okamura et al. found that insulin sensitivity increased after significant improvement of depression due to different antidepressants (*n* = 20) [29–36]. This limits the accuracy of potential comparisons of our results to those previously reported, especially given that IR is a state-marker of MDD. On the other hand, our work has reinforced the observations made by Rashidian et al., who noted that in a prospective study in MDD patients (*n* = 79) treated with vortioxetine IR was linked to non-response. This association remained significant after controlling for baseline BMI and several other factors, such as depression severity, serum lipid profile and C-reactive protein (CRP) [19]. This could suggest that IR is linked to non-response to antidepressants in MDD regardless of their mechanisms of action. Perhaps IR should be taken into consideration in the case of non-response to antidepressants, and efforts should be made to develop and implement insulin-sensitizing interventions such as physical activity/ lifestyle-focused programs instead of drug switches or augmentation [7].

Furthermore, in this work, significant differences were noted between MDD T[-] vs. MDD [+] regarding psychopathology, that is, higher severity of: (1) depressive symptom domains: depressed mood, appetite/ weight, general interest, energy level and overall severity of depression assessed by QIDS and HADS with medium to large effect sizes, (2) anhedonia as measured with total SHAPS and DARS subscale of hobbies with large effect sizes, (3) anxiety as assessed with STAI state and trait subscales with large effect sizes, (4) daytime dysfunction and overall impairment of sleep quality evaluated with PSQI with large effect sizes. A significantly higher level of bipolar spectrum features was noted in MDD T [-] vs. HC on MDQ but not on HCL. Understandably so, as these tools are dissimilar [37], it was previously reported that in Polish versions of these tools, MDQ has higher sensitivity than HCL [38]. Noteworthy, no differences between MDD T[+] and MDD T[-] were found regarding the level of bipolar spectrum features, which might limit the effectiveness of antidepressants in monotherapy [39] and could potentially influence our results. While it seems obvious that patients non-responsive to treatment would report more severe depressive symptoms, it is interesting that not all symptoms differentiate between MDD T[-] and MDD T[+]. We showed that the symptoms linked to non-response were depressed mood, changes in appetite/ weight, loss of interest (related to the motivational component of anhedonia), overall anhedonia and its hobbies component, energy level, state and trait anxiety, and impairment of sleep. Next, significant associations were noted between the levels of insulin and HOMA-IR and all these symptoms and several others: psychomotor agitation/ retardation, thoughts of death/ suicide, subjective sleep quality, and use of sleep medication. This would suggest that non-responsive patients could be characterized by a distinct “metabolic” presentation of depression, which is linked to IR. This hypothesis would be in congruence with available data [16, 17, 40]. Similarly to us, Brouwer et al. reported that MDD subjects comorbid with type 2 diabetes (TMD2) with higher IR reported more pronounced irritability, anhedonia (loss of interest, loss of pleasure and enjoyment), energy level/ fatigue, and hypersomnia. They also concluded that these subjects benefited from light therapy more than those with low IR [16]. Shell et al. analyzed middle-aged MDD individuals with elevated cardiovascular risk (hypertension, hypercholesterolemia, or nicotine SUD), of which the majority were in the prediabetes stage, to find associations between somatic symptoms of MDD and metabolic as well as inflammatory factors. In accordance with our results, they found that IR was significantly linked to changes in appetite and hypersomnia. Contrary to our observations, they noted that BMI was associated with increased appetite and hypersomnia, BMI partially mediated the links between IR and increased appetite and hypersomnia. Interestingly, some of these relationships were moderated by race [17]. Moreover, Chae et al. explored the associations between particular depressive symptoms and metabolic markers. Likewise, their work indicated several positive links between insulin and increased appetite, hypersomnia, and insomnia, as well as suicidal thoughts. In a reverse analysis (markers as independent variables and symptoms as dependent variables), they noted strong links between insulin and depressed mood as well as loss of interest. While they reported several other significant connections between immunometabolic markers (glucose, lipids, high-sensitivity CRP) and depressive symptoms, they concluded that BMI only contributed to a small part of these [40]. Also, in a large sample of participants based on the Netherlands Study of Depression and Anxiety, Van Haeringen et al. conducted a proteomic analysis. They found significant associations between the network of proteins involved in inflammatory and metabolic processes and specific depressive symptoms, mainly changes in appetite/ weight, sleep, and panic. Once corrected for BMI, none of the results remained significant. Still, the authors concluded that due to the complex role of BMI in the connections between MDD and inflammation, such corrections might lead to overadjustment [41]. Moreover, correspondingly to Valsamakis et al., who highlighted the possible role of hypothalamic–pituitary–adrenal axis in this association, we observed that state and trait anxiety are linked to IR. We also noted that higher state and trait anxiety were associated with non-response to SNRI [42].

Several limitations of our work need to be acknowledged. Firstly, the cross-sectional design which does not allow to draw any causal relationships between IR and non-response to SNRI or specific MDD symptoms. Secondly, the small study sample which might have resulted in insufficient power of this study to find all relevant links between studied variables. Nonetheless, our study included a larger group of MDD subjects than most of the already available, which reported on the associations between insulin, IR, and treatment response to antidepressants. In this study, CGI-I was as the measure of treatment response. It could be argued that the use of a depression-specific measure such as the Hamilton Depression Rating Scale (HAM-D) would be preferred, which used to be the “gold standard” measure of depression in the past [43]. Yet this would require a different longitudinal methodology, while as stated in the materials and methods section, this was a cross-sectional study. Moreover, the majority of current definitions of treatment-resistant MDD do not specify the outcome measure tools suitable for the assessment of treatment response [44]. Notably, it was shown that CGI-I is rather commonly used as the outcome measure in studies of MDD [45], and its results correlate well with HAM-D [46]. Next, the choice of the SNRI and its’ dose relied on the attending physician’s choice. In the majority of patients receiving SNRI, the dose was raised to the maximal recommended within the SNRI range (venlafaxine 225 mg/d or higher, duloxetine 120 mg/d) before deeming the patient non-responsive. Still, some patients would not tolerate it due to adverse effects or wished to change to another antidepressant and we respected that. This could result in potential pseudo-non-response in some patients (e.g., due to lower serum levels of duloxetine in smokers *n* = 6) [47]. These issues pertain to the cross-sectional methodology and the “real-world” character of this work, and there is definitely a need for further evaluation of the relationship between IR and MDD in prospective trials with a more robust methodology [48].

Still, our work constitutes a meaningful contribution to the current knowledge on links between IR and MDD. This was the first study to show that IR is positively associated with non-response to SNRI in MDD. Furthermore, we noted that SNRI-responsive and non-responsive patients were dissimilar in MDD clinical presentation and that most of the symptoms differing between subjects responsive and non-responsive to SNRI were significantly associated with insulin and IR (but not BMI). Interestingly, we found no links between BMI and particular MDD symptoms, possibly due to a small study sample.

## Conclusions

The obtained results indicate that insulin and IR are significantly associated with a particular depressive clinical presentation, anhedonia, state and trait anxiety and impaired sleep as well as non-response to SNRI.

## Data Availability

The datasets generated during and/or analyzed during the current study are available from the corresponding author upon reasonable request.
